# Diagnostic Performance of an Automated System for Assaying Anti-Hepatitis E Virus Immunoglobulins M and G Compared with a Conventional Microplate Assay

**DOI:** 10.3390/v14051065

**Published:** 2022-05-17

**Authors:** Florence Abravanel, Delphine Parraud, Sabine Chapuy-Regaud, Marcel Miedouge, Estelle Bonnin, Margaux Larrieu, Alexandre Aversenq, Sébastien Lhomme, Jacques Izopet

**Affiliations:** 1 CHU Toulouse, Hôpital Purpan, Laboratoire de Virologie, National Reference Center for Hepatitis E, 31300 Toulouse, France; delphine.parraud@laposte.net (D.P.); chapuy-regaud.s@chu-toulouse.fr (S.C.-R.); miedouge.m@chu-toulouse.fr (M.M.); bonnin.e@chu-toulouse.fr (E.B.); larrieu.m@chu-toulouse.fr (M.L.); aversenq.a@chu-toulouse.fr (A.A.); lhomme.s@chu-toulouse.fr (S.L.); izopet.j@chu-toulouse.fr (J.I.); 2 Inserm UMR 1291, CNRS UMR5051, Université Toulouse III, 31000 Toulouse, France

**Keywords:** hepatitis E virus, serology, immunocompetent patients, immunocompromised patients

## Abstract

To evaluate the diagnostic performance of the Liaison^®^ Murex anti-HEV IgM and IgG assays running on the Liaison^®^ instrument and compare the results with those obtained with Wantai HEV assays. We tested samples collected in immunocompetent and immunocompromised patients during the acute (HEV RNA positive, anti-HEV IgM positive) and the post-viremic phase (HEV RNA negative, anti-HEV IgM positive) of infections. The specificity was assessed by testing HEV RNA negative/anti-HEV IgG-IgM negative samples. The clinical sensitivity of the Liaison^®^ IgM assay was 100% for acute-phase samples (56/56) and 57.4% (27/47) for post-viremic samples from immunocompetent patients. It was 93.8% (30/32) for acute-phase (viremic) samples and 71%% (22/31) for post-viremic samples from immunocompromised patients. The clinical sensitivity of the Liaison^®^ IgG assay was 100% for viremic samples (56/56) and 94.6% (43/47) for post-viremic samples from immunocompetent patients. It was 84.3% (27/32) for viremic samples and 93.5% (29/31) for post-viremic samples from immunocompromised patients. Specificity was very high (>99%) in both populations. We checked the limit of detection stated for the Liaison^®^ IgG assay (0.3 U/mL). The clinical performance of the Liaison^®^ ANTI-HEV assays was good. These rapid, automated assays for detecting anti-HEV antibodies will greatly enhance the arsenal for diagnosing HEV infections.

## 1. Introduction

The hepatitis E virus (HEV) is a leading cause of acute hepatitis worldwide. According to the WHO, at least 20 million hepatitis E virus (HEV) infections occur annually [1]. HEV genome RNA possesses a 5′ 7-methylguanosine cap structure followed by a short 5′ untranslated region (UTR); three major open reading frames ([ORFs]: ORF1, ORF2, and ORF3); and a 3′UTR. ORF1 is the largest viral gene product of HEV and contains the nonstructural replication machinery of the virus. ORF2, which is located downstream of ORF1 encodes the viral capsid protein. ORF3 encodes a small protein required for the release of virions [1]. HEV is the only member of the *Hepeviridae* family, subfamily *Orthohepevirinae*. The genus *Paslahepevirus* has two species: (*balayani* and *alci*)). Most human infections are with the species *Paslahepevirus balayani* genotypes 1, 2, 3, and 4 and less frequently 7. Genotypes 1 and 2 are limited to humans and mostly affect developing countries. In industrialized countries, clinical cases of HEV genotypes 3 and 4 tend to be more sporadic, with zoonotic transmission via direct contact with infected animals or consumption of contaminated animal meats. [1]. The picture is complicated by recent reports of a rat HEV, a phylogenetically distinct genus with the *Hepeviridae* family and the *Rocahepevirus* genotype C1 that causes severe hepatitis in immunocompetent and immunocompromised patients [2,3,4,5].

Most HEV infections are self-limiting in immunocompetent patients [6,7], but chronic infections, in which the virus persists for more than three months [6], can occur in immunocompromised patients, such as solid organ transplant recipients, patients with hematological disease on chemotherapy, stem cell transplant recipients, and patients co-infected with HIV with low T CD4 + counts [6,7]. The clinical manifestations can range from typical acute hepatitis to extrahepatic disorders. Neurological and renal manifestations are the most consistently reported and include neuralgic amyotrophy, Guillain–Barré syndrome, acute kidney injury, and glomerular disease [7,8,9].

For laboratory diagnosis, detection of HEV antibodies provides a good understanding of HEV infection and aids in diagnosis. The European Association for the Study of the Liver (EASL) recommends using a combination of serology and HEV RNA [10]. HEV RNA can be detected very early in the acute course of infection and starts to decline in serum to undetectable levels 7–10 weeks post infection [11,12]. RNA is detectable only during the acute phase of infection, making it more specific to acute infection than Anti-HEV IgM detection, which also takes place during both the acute and the convalescent periods. [13]. An anti-HEV IgM test is performed first if an HEV infection is suspected because the currently available assays perform well [13]. HEV RNA testing is essential for diagnosing infections in immunocompromised patients because anti-HEV IgM antibodies may be absent due to immunosuppression.

There are several serological assays for HEV antibodies, but only two of them have been automated: the Vidas^®^ assays and the Virclia^®^ assays. However, Diasorin has recently released two serological assays that will run on Liaison^®^ instruments. We evaluated the clinical performance of the new Liaison^®^ MUREX anti-HEV IgG and IgM assays by testing samples from immunocompetent and immunocompromised patients.

## 2. Materials and Methods

### 2.1. Patients

We tested 438 samples from immunocompetent and immunocompromised patients (mean age: 48 years; range 5–87 years; male/female ratio: 1.1) characterized for hepatitis E (HEV RNA detection and Wantai HEV IgM assay). Data were analysed with reference to the infectious profile defined with the serological result using Wantai assays and the HEV RNA results. Data included samples from the viremic phase (Wantai HEV IgM and HEV RNA positive; *n* = 88; 56 and 32 collected in immunocompetent and immunocompromised patients respectively), the post-viremic phase (Wantai IgM positive, HEV RNA negative; *n* = 78, 47 and 31 collected in immunocompetent and immunocompromised patients respectively), and the uninfected (HEV RNA negative and Wantai HEV IgG and IgM negative; *n* = 272, 207 and 65 collected in immunocompetent and immunocompromised patients respectively). The samples were collected between September 2016 and June 2017 during hospitalization and routine out-patient visits and stored frozen. Patient records and information were anonymized and de-identified prior to analysis. We also tested patients infected with HEV genotype 1 (*n* = 4), genotype 2 (*n* = 2), and genotype 4 (*n* = 5) collected at the acute phase (HEV RNA positive). We estimated the limit of detection of the Liaison^®^ Murex IgG assay by testing serial 2-fold dilutions of a clinical HEV IgG-positive sample, quantified using the Wantai HEV IgG assay [14], on the Liaison^®^ Murex system. Ethical approval was obtained from the French *Comité de Protection des Personnes* Nord Ouest I (N°2017-A00196-47).

### 2.2. Methods

The Liaison^®^ Murex HEV IgG and IgM assays (Diasorin, Saluggia, Italy) are CE certified. They use 100 µL samples (blood plasma or serum). Both these immunoluminescent immunoassays were coated with genotype 1 and 3 ORF2 antigens. Values obtained with the qualitative Liaison^®^ Murex HEV IgM assay are automatically calculated by the instrument as an index. Samples with an IgM index value below the threshold value (set at 1.00) were considered to be negative.

The quantitative Liaison^®^ Murex HEV IgG measures the intensity of the luminescence, which is proportional to the concentration of HEV IgG in the sample. Test values are automatically calculated by the instrument and expressed as IU/mL. The Liaison^®^ Murex HEV IgG assay has been standardized indirectly against the WHO international standard NIBSC 95/584. IgG values below the threshold value (0.3 IU/mL) were considered to be negative. The IgG test is linear up to 10 IU/mL.

The Wantai HEV IgG and IgM EIA kits (Wantai Biologic Pharmacy Enterprise, Beijing, China, certified CE) were used according to the manufacturer’s instructions. HEV RNA was tested using an accredited ISO15189 RT-PCR assay that targets the ORF3 region [15].

## 3. Results

### 3.1. Clinical Performance of the Liaison^®^ Murex Anti-HEV IgM Assay

#### 3.1.1. Specificity and Sensitivity of the Liaison^®^ Murex ANTI-HEV IgM in Immunocompetent Patients

**Figure 1:**
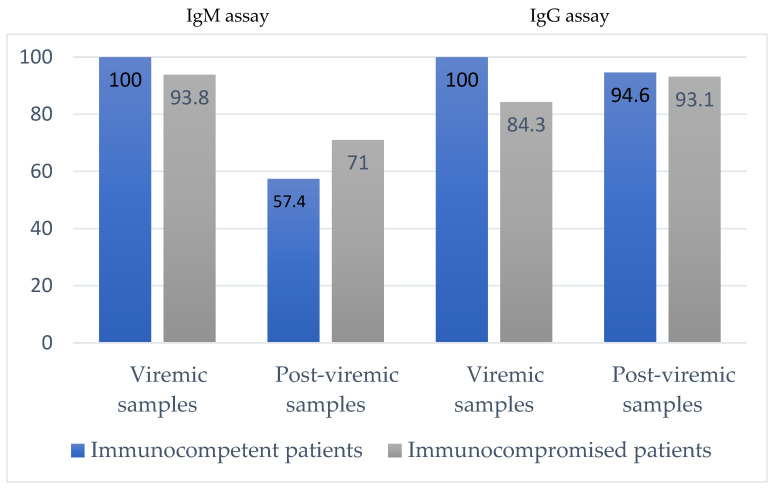
Clinical sensitivities of the Liaison^®^ Murex assays in immunocompetent and immunocompromised patients.

None of the 207 negative samples tested positive with the Liaison^®^ anti-HEV IgM (specificity: 100%; 95% CI: 98.55–100%). All 56 acute-phase samples (viremic samples) tested positive with the Liaison^®^ anti-HEV IgM assay (sensitivity: 100%; 95% CI: 94.6–100%) (Figure 1). The 47 Wantai IgM-positive HEV RNA-negative samples (post-viremic) included 27 positive and 20 negative samples (sensitivity: 57.4%; 95% CI: 43.3–0.71.6%). The median Wantai signal-to-cutoff ratios of the 20 Wantai IgM-positive and Liaison IgM-negative samples was lower (median = 2.4) than the median Wantai signal-to-cutoff ratios of the 20 IgM Wantai and Liaison^®^ positive samples (median = 3.2, *p* = 0.02). The 20 samples that tested negative with the Liaison^®^ anti-HEV IgM assay included 16 that were positive with both the Liaison^®^ anti-HEV IgG and Wantai anti-HEV IgG assays.

#### 3.1.2. Specificity and Sensitivity of the Liaison^®^ Murex ANTI-HEV IgM in Immunocompromised Patients

All 65 of the negative samples tested negative with the Liaison^®^ assay (specificity: 100%; 95% CI: 95.4–100%) (Figure 1). The 32 acute-phase samples included 30 that tested positive with the Liaison^®^ ANTI-HEV IgM assay (sensitivity: 93.75%; 95% CI: 85.4–100%). The 31 Wantai IgM-positive/HEV RNA-negative samples included 22 that tested positive and 9 that tested negative with the Liaison^®^ IgM assay (sensitivity: 71%; 95% CI: 55–86.9%). The 9 Liaison^®^ IgM-negative samples included 7 that were positive with the Liaison^®^ IgG and Wantai HEV IgG assays.

### 3.2. Clinical Performance of the Liaison^®^ Murex Anti-HEV IgG Assay

#### 3.2.1. Specificity and Sensitivity of the Liaison^®^ Murex ANTI-HEV IgG in Immunocompetent Patients

Two of the 207 negative samples tested positive with the Liaison^®^ IgG assay (specificity: 99%; 95% CI: 97.6–100%), while all 56 viremic samples tested positive with the Liaison^®^ IgG assay (sensitivity: 100%; 95% CI: 94.64–100%) (Figure 1). We note that only 51/56 (91.1%) were IgG positive with the Wantai assay. Similarly, 43/47 post-viremic samples tested positive (sensitivity: 91.55%; 95% CI: 85.5–99.5%). The 4 negative samples also tested negative with the Wantai IgG assay.

#### 3.2.2. Specificity and Sensitivity of the Liaison^®^ Murex ANTI-HEV IgG in Immunocompromised Patients

All 65 negative samples were negative with the Liaison^®^ IgG assay (specificity: 100%; 95% CI: 95.4–100%), but 27/32 viremic phase samples tested positive with the Liaison^®^ ANTI-HEV IgG assay (sensitivity: 84.3%; 95% CI: 71.8–96.97%) (Figure 1). Note that only 25/32 (78%) were IgG positive with the Wantai assay, while 29/31 post-viremic samples tested positive with both the Liaison^®^ and the Wantai IgG assays (sensitivity: 93.5%; 95% CI: 84.9–100%).

### 3.3. Evaluation of the Limit of Detection of the Liaison^®^ Murex ANTI-HEV IgG

**Figure 2:**
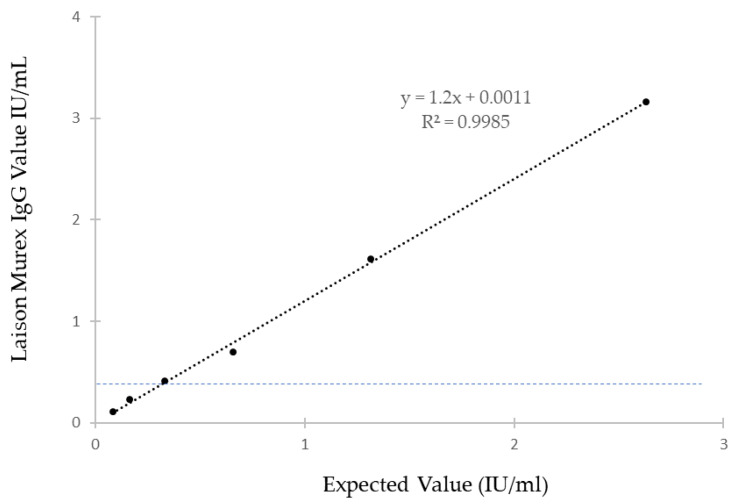
Correlation between the expected IgG HEV titers and the Liaison^®^ IgG HEV results. The dashed line represents the limit of detection of the Liaison^®^ Murex IgG HEV assay.

The HEV IgG-positive sample quantified with the Wantai anti-HEV IgG assay (2.63 IU/mL) was serially diluted and tested with the Liaison^®^ assay (Table 1). The greatest dilution that tested positive with the Liaison^®^ HEV IgG assay was 0.33 IU/mL. The correlation between the expected values and the Liaison ^®^ anti-HEV IgG Murex values was good (Figure 2).

### 3.4. HEV Antibody Detection in HEV Genotype 1, 2 and 4 Infections

All the samples from patients with HEV genotype 1 or 2 infections tested positive for HEV IgG and IgM using both the Wantai and Liaison^®^ assays. Most (4/5) of the samples from patients with HEV genotype 4 tested positive for HEV IgG and IgM using both the Wantai and Liaison^®^ assays, while one was HEV IgG positive and HEV IgM negative with both assays, suggesting a reinfection.

## 4. Discussion

Our laboratory evaluation of HEV IgM and HEV IgG assays developed for the Liaison^®^ instrument indicated that they are suitable for diagnosing acute HEV infections.

HEV serological assays are relatively easy to perform and less expensive than molecular tests, but they vary in performance characteristics with regard to sensitivity and specificity, which has complicated data interpretation [16,17,18,19,20]. We find that the Liaison^®^ IgM assay is very sensitive (100%) with samples from acutely infected (HEV RNA positive) immunocompetent patients and less so (93.75%) with those from immunocompromised patients. However, the Liaison^®^ IgM assay was less sensitive (only 59.15%) when tested on Wantai HEV IgM-positive/HEV RNA-negative samples from immunocompetent patients. This ability to detect long-lasting HEV IgM may vary with the serological assay [21]. We have previously reported that the VIDAS IgM assay is less effective than the Wantai assay for detecting low concentrations of HEV IgM [22]. The clinical sensitivity of the VIDAS IgM assay was 97.65% for viremic samples (83/85) and 59.15% (42/71) for post-viremic samples from immunocompetent patients. It was 78.95% (45/57) for acute phase samples and 77.78% (28/36) for post-viremic samples from immunocompromised patients [22]. Norder et al. found that the DiaPro assay remained positive for the longest time after the onset of an HEV infection [17]. Riveiro-Barciela et al. showed that the persistence of HEV IgM was 56% using Mikrogen anti-HEV IgM, while it was 24% using Wantai anti-HEV IgM at a median time of 34 months after an episode of acute hepatitis E [21]. The cut-off value determined by the manufacturer may also influence assay sensitivity [20]. This restricted ability to detect long-lasting HEV IgM is an advantage for the diagnosis of recent HEV infections, as the clinician can rely on the HEV IgM assay to diagnose an acute hepatitis E infection. The persistence of IgM detection raises the question of which HEV antigen detection is a better serological marker for diagnosing a hepatitis E infection [21]. However, there is only one antigen assay available, and it is a microplate assay; thus antigen tests are more frequently performed by batch. The antigen test’s diagnostic sensitivity for an acute HEV infection was 91%, with no significant difference between immunocompetent (88%) and immunocompromised (94%) patients [23]. A fully automated random-access IgM assay is more efficient for rapidly diagnosing acute hepatitis E.

The analytical sensitivity of HEV IgG assays also varies, which strongly influences the seroprevalence rates in epidemiological studies [19,24,25,26]. Evaluations of the analytical sensitivity of HEV IgG assays have found differences of from 0.2 U/mL (Wantai assay) to 6.31 U/mL (Mikrogen assay, old version) [17,20,27]. We did not evaluate the limit of detection of the Liaison ^®^ IgG assay using the WHO standard (NIBSC code: 95/584); we used a clinical sample whose HEV IgG concentration was determined using the Wantai assay [14]. Our results are in agreement with the limit of detection stated for the Liaison^®^ IgG assay (0.3 U/mL). Thus, we find that the Liaison^®^ IgG assay is a suitable tool for seroprevalence studies.

We also assessed the capacity of the Liaison^®^ assays to detect anti-HEV antibodies in patients infected with genotypes 1, 2, or 4. We recognize that only a few samples were tested. A greater number of samples need to be tested to certify the efficiency of detection of multiple genotypes. However, the Liaison^®^ assays can detect HEV antibodies with the same spectrum as the Wantai assay. This is consistent with HEV having a single serotype [28].

Commercial test formats are typically designed for batch-wise processing, relying on techniques such as ELISA [13]. However, robust, easy-to-use, fast assays are required for the special emergency setting of acute liver failure. Few HEV serological assays have been implemented with random access automatic systems. Two automated HEV serological assays are on the market: the Vidas ^®^ and the Virclia ^®^ instruments. We previously evaluated the Vidas^®^ anti-HEV IgG and IgM assays (Biomérieux, Marcy-l’Étoile, France); their specificity and sensitivity were excellent [22]. Similarly, the novel Virclia monotest^®^ (VirCell, Granada, Spain) hepatitis E IgM and IgG assay is very sensitive for both immunoglobulins (100% for 20 HEV RNA positive samples), but its specificity was not assessed on a large panel of negative samples [29].

We therefore conclude that the Liaison^®^ Murex assays are very sensitive and specific. They are suitable for diagnosing acute HEV infections, particularly in immunocompetent patients. The Liaison instrument allows simple, efficient sample handling, making the assays particularly suitable for diagnosis in emergency settings and for high-throughput laboratories.

## Figures and Tables

**Table 1:** Evaluation of the limit of detection of the Liaison^®^ Murex IgG assay: Results of the serial 2-fold dilutions of an HEV IgG-positive sample.

Expected Value (IU/mL)	Murex IgG HEV Quantification(IU/mL)	Murex IgG HEV Interpretation
2.63	3.16	positive
1.32	1.61	positive
0.66	0.7	positive
0.33	0.41	positive
0.16	0.23	negative
0.08	0.11	negative

## Data Availability

The data presented in this study are available on request from the corresponding author.
